# Chimerism in piglets developed from aggregated cloned embryos

**DOI:** 10.1002/2211-5463.12037

**Published:** 2016-03-15

**Authors:** Yongye Huang, Zhanjun Li, Anfeng Wang, Xiaolei Han, Yuning Song, Lin Yuan, Tianye Li, Bing Wang, Liangxue Lai, Hongsheng Ouyang, Daxin Pang

**Affiliations:** ^1^ College of Life and Health SciencesNortheastern UniversityShenyangChina; ^2^ Jilin Provincial Key Laboratory of Animal Embryo EngineeringCollege of Animal SciencesJilin UniversityChangchunChina

**Keywords:** aggregation, chimerism, development, somatic cell nuclear transfer

## Abstract

Porcine chimeras are valuable in the study of pluripotency, embryogenesis and development. It would be meaningful to generate chimeric piglets from somatic cell nuclear transfer embryos. In this study, two cell lines expressing the fluorescent markers enhanced green fluorescent protein (EGFP) and tdTomato were used as donor cells to produce reconstructed embryos. Chimeric embryos were generated by aggregating two EGFP‐cell derived embryos with two tdTomato‐cell derived embryos at the 4‐cell stage, and embryo transfer was performed when the aggregated embryos developed into blastocysts. Live porcine chimeras were successfully born and chimerism was observed by their skin color, gene integration, microsatellite loci composition and fluorescent protein expression. The chimeric piglets were largely composed of EGFP‐expressing cells, and this phenomenon was possibly due to the hyper‐methylation of the promoter of the tdTomato gene. In addition, the expression levels of tumorigenicity‐related genes were altered after tdTomato transfection in bladder cancer cells. The results show that chimeric pigs can be produced by aggregating cloned embryos and that the developmental capability of the cloned embryo in the subsequent chimeric development could be affected by the growth characteristics of its donor cell.

**Abbreviations**
CCK‐8: Cell Counting Kit‐8
CMV: cytomegalovirus
COCs: cumulus‐oocyte complexes
DMEM: Dulbecco's Modified Eagle's Medium
EF1α: elongation factor 1α
EGFP: enhanced green fluorescent protein
ER: endoplasmic reticulum
FBS: fetal bovine serum
GRP78: glucose regulated protein, 78 kDa
IRE1: inositol‐requiring enzyme 1
Oct4: octamer‐binding protein 4
PBS: phosphate‐buffered saline
PCR: polymerase chain reaction
PHA: phytohemagglutinin
PZM3: zygote medium‐3
SCNT: somatic cell nuclear transfer
VPA: valproic acid

A hallmark of the pluripotency of embryonic stem (ES) cells and induced pluripotent stem (iPS) cells was demonstrated by the observation that their subclonal cultures are distributed into all embryonic tissues including germ cells when reintroduced into other host embryos 1, 2. In addition to the investigation of embryo pluripotency characteristics, chimeric embryos are also valuable in studies focused on embryogenesis and fetogenesis 3, 4, 5 and in transgenic research 4, 6, 7, 8. However, reports on porcine chimeras were rare. In one previous report, four piglets were found to have a small degree of pigmentation chimerism when embryonic germ (EG) cells were injected into the host embryos, but microsatellite analysis failed to confirm this 9. For iPS cells, chimerism was only observed in limited areas in a fetus at day 65 of gestation 10. High coat color chimerism was observed when producing chimeric pigs by inner cell mass injection into *in vivo* blastocysts 11, but it is costly and inconvenient to harvest chimeras using this method. Somatic cell nuclear transfer (SCNT) combined with genetic modification is useful in various aspects. Until recently, live porcine chimeras were only harvested from SCNT embryos by blastomere injection 12. The toughness of the pig trophectoderm was thought to be an obstacle for chimeric pig production 13. Therefore, another simple and effective method for producing porcine chimeras should be determined.

In the rhesus monkey, the aggregation of totipotent cells at the 4‐cell stage in embryos was considered to be an effective method for the production of chimeric offspring 14. The method of generating embryo––embryo chimeras has been described previously 15. In pigs, chimeric fetuses have also been generated from the aggregation of inner cell mass cells and parthenogenetic embryos, although the gestation was artificially terminated at the somite stage 16. In another report, cloned miniature pigs were generated by the aggregation of handmade cloned embryos at the 4‐cell stage 17. In this study, we attempt to generate live porcine chimeras by aggregating SCNT embryos derived from donor cells with expression of different fluorescent proteins.

The competition and cooperation among cells in the embryogenesis of multicellular organisms is an interesting research topic, and a network of genes associated with cell competition has been identified 18, 19. Cellular and/or molecular changes after aggregation would exacerbate the competition within chimeric embryos, thus leading to varied composition rate during the subsequent chimeric development. Therefore, the mechanism underlying chimeric development should be widely exploited.

## Materials and methods

### Ethics statement

All animal experiments were conducted according to the guidelines on animal care and use established by the Animal Care and Welfare Committee of Jilin University, with the approval number 2011‐036.

### Animals

Porcine ovaries were provided by the HuaZheng Agriculture Development Co., Ltd., which is located in Changchun City, and permission for usage was also obtained from the company. Embryo transfer and pig farming were carried out at HuiChang Livestock Co., Ltd. (located in Changchun City, China).

### Chemicals and reagents

Unless otherwise indicated, all chemicals were purchased from Sigma Chemical Co. (St. Louis, MO, USA). All of the solutions and media were filtered using a 0.22‐mm filter. The cell culture medium was composed of Dulbecco's Modified Eagle's Medium (DMEM; Gibco, Grand Island, NY, USA) plus 10% fetal bovine serum (FBS; PAA, Pasching, Austria), non‐essential amino acid (NEAA; PAA), glutamine (PAA), and penicillin/streptomycin (PAA). The cells were frozen with FBS containing 10% DMSO. The maturation media consisted of TCM 199, 0.1% polyvinyl alcohol, cysteine (0.1 mg·mL^−1^), epidermal growth factor (10 ng·mL^−1^), 0.91 mm Na‐pyruvate, 3.05 mm d‐glucose, follicle‐stimulating hormone (0.5 mg·mL^−1^), luteinizing hormone (0.5 mg·mL^−1^), penicillin (75 mg·mL^−1^) and streptomycin (50 mg·mL^−1^). The reconstructed embryos were fused in medium containing 0.3 m mannitol, 1.0 mm CaCl_2_.2H_2_O, 1.0 mm MgCl_2_.6H_2_O, and 0.5 mm Hepes. The embryo culture medium was porcine zygote medium 3 (PZM3) consisting of 108.0 mmol·L^−1^ NaCl, 10.0 mmol·L^−1^ KCl, 0.35 mmol·L^−1^ KH2PO4, 0.4 mmol·L^−1^ MgSO4.7H2O, 25.07 mmol·L^−1^ NaHCO3, 0.2 mmol·L^−1^ sodium‐pyruvate, 2.0 mmol·L^−1^ Ca(lactate)2.5H2O, 1.0 mmol·L^−1^ glutamine, 5.0 mmol·L^−1^ hypotaurine, 20 mL·L^−1^ Eagle's basal medium amino acid solution, 10 mL·L^−1^ modified Eagle's medium amino acid solution, 75 mg·mL^−1^ penicillin, 50 mg·mL^−1^ streptomycin, and 3 mg·mL^−1^ bovine serum albumin (PAA).

### Preparation of donor cells for SCNT

Fetal fibroblast cells were harvested from 33‐ to 35‐day‐old fetuses, and the preparation was performed as previously described 20. Next, transgenic porcine fetal fibroblast cells were produced. The pEF1α‐EGFP vector was constructed from the pIRES2‐EGFP vector (Clontech, Palo Alto, CA, USA) with the EGFP gene being regulated by an EF1α promoter; and the pCMV‐tdTomato vector was purchased (Clontech). After linearizing the vectors, cell transfection was performed using the FuGENE HD transfection reagent (Roche, Mannheim, Germany) according to the manufacturer's protocol. The cells were split 1 : 20 into fresh cell culture medium 24 h after transfection, and starting 48 h later, cells were selected with 500 μg·mL^−1^ of G418 (AMERCO) for an additional 7 days. After selection with G418, cell colonies were obtained by fluorescence detection with a fluorescence microscope. The positive cell colonies were maintained and propagated. Once they were grown to confluence, the cells were frozen for further use. Before nuclear transfer, the cells were thawed and cultured. Single cell suspensions were prepared by trypsinization prior to somatic cell nuclear transfer (SCNT). Except for those cells sent for SCNT, the rest of the cells were cultured and refrozen for further use after reaching confluence.

### Cell proliferation assay

Cell proliferation was measured with the use of a Cell Counting Kit‐8 (Dojindo, Gaithersburg, MD, USA). Briefly, cells were plated in 96‐well plates at a density of 2000 per well in 100 μL cell culture media. The cells were incubated at 39 °C in a 5% CO_2_ humidified atmosphere for 24, 48, 72 or 96 h, and 10 μL CCK‐8 solution was added to the cells. After incubation for 1 h, the absorbance at 450 nm was measured using a microplate reader (Tecan, Groedig, Austria).

### Cell cycle analysis

Cell cycle analysis was conducted using a Cell Cycle Detection Kit (KeyGEN Biotech, Nanjing, China) according to the manufacturer's instructions. Briefly, cells were washed with phosphate‐buffered saline (PBS) buffer and centrifuged at 100 ***g*** for 5 min. After the supernatant was removed, the cells were adjusted to a density of 1 × 10^6^ cells·mL^−1^. Next, cells were fixed with 70% ethanol at 4 °C overnight. Following a wash with PBS, the cell pellet was resuspended in 100 μL of RNase A at 4 °C for 30 min. An additional 400 μL of propidium iodide buffer was added, and the cells were stained at 4 °C for another 30 min. Finally, the analysis was performed using a BD FACSCalibur flow cytometer (Becton Dickinson Immunocytometry Systems, Franklin Lakes, NJ, USA).

### Somatic cell nuclear transfer and aggregation of embryos at the 4‐cell stage

The generation of SCNT embryos was performed as previously described 21. Briefly, ovaries were collected at a local slaughterhouse and transported to the laboratory in saline at 32–37 °C. The follicular contents were recovered by aspiration of 3 to 6 mm follicles using an 18‐gauge needle and a 10‐mL disposable syringe. Only cumulus‐oocyte complexes (COCs) with uniform cytoplasm and at least three layers of cumulus cells were selected. After rinsing three times, COCs were transferred to a 35‐mm dish containing 2 mL of maturation medium covered with mineral oil, then COCs were matured at 39 °C in a 5% CO_2_ atmosphere.

After maturation for 42–44 h, COCs were denuded with 0.1% hyaluronidase. Only the oocytes with an extruded first polar body with intact cytoplasm and a round shape were used as the recipients of SCNT. A single donor cell with the proper size and good morphology was injected into the perivitelline space and placed adjacent to the recipient cytoplasm. Consequently, the fusion and activation of cell‐cytoplast complexes was accomplished with 2 DC pulses of 1.2 KV·cm^−1^ for 30 μs using a BTX Electro Cell Manipulator 2001 (BTX, San Diego, CA, USA) in a chamber filled with fusion medium. Next, the reconstructed embryos were washed and incubated in porcine zygote medium‐3 (PZM3) and cultured at 39 °C in 5% CO_2_ humidified air.

Following activation, after 48–52 h, SCNT embryos that had developed into the 4‐cell stage were selected. The zona pellucidae of embryos were removed by treatment with 3.3 mg·mL^−1^ pronase solution. Embryos derived from EGFP‐ and tdTomato‐expressing cells were aggregated together by being mechanically pushed against each other in a droplet of 1 mg·mL^−1^ phytohemagglutinin (PHA). After washing with PZM3, an aggregated embryo was transferred into one 15 μL PZM3 drop, and further culturing was performed at 39 °C in 5% CO_2_ humidified air.

### Embryo transfer

The aggregated blastocysts with good morphology were transferred to the uterine horns of naturally cycling surrogate sows on day 5 or day 6 of standing estrus. The injected embryos were transferred to the uterine horns of naturally cycling surrogate sows on day 3 of standing estrus on the following day after injection. Pregnancy was diagnosed by ultrasonography examination on day 27 after SCNT and then checked regularly at 2 week intervals.

### DNA isolation, polymerase chain reaction (PCR), and microsatellite analysis

To detect the chimerism in porcine off spring, genomic DNA was extracted using the TIANmp Genomic DNA Kit (TIANGEN, Beijing, China). The EGFP, tdTomato, and SRY gene were amplified using PCR analysis (see Table 1 for primer sequences and PCR conditions).

**Table 1:** Primer sequences for PCR analysis

Genes	Primer sequences (5′–3′)	Length (bp)	*T*_m_ (°C)
GFP	F: CAG TGC TTC AGC CGC TAC CC R: TGC CGT TCT TCT GCT TGT CG	277	58
tdTomato	F: AGG GCG AGG AGG TCA TCA AA R: CAT GGT CTT CTT CTG CAT TAC GG	416	58
SRY	F: GCT TTC ATT GTG TGG TCT CGT R: CTT GGC GAC TGT GTA TGT GAA G	309	58
GAPDH	F: GAT GGC CCC TCT GGG AAA CTG TG R: GGA CGC CTG CTT CAC CAC CTT CT	404	58
EF1α outside	F: GCG TTT TAG CGT ATA TGT TCG GCG A R: TCA CGA CAC CTA AAA TAA AAA AAA	558	53
EF1α inside	F: TTG TTG TAG GGA GTT TAA AAT GGA G R: CCA CCC ACT CAA TAT AAA AAA ACT C	231	52
CMV outside	F: GTT TGG TTG ATC GTT TAA CGA TTT TC R: AAC GAT TCA CTA AAC CAA CTC TAC TT	497	53
CMV inside	F: TGA TTT TAT GGG ATT TTT TTA TTT G R: ATT CAC TAA ACC AAC TCT ACT TAT ATA AAC	278	52

Microsatellite analysis was performed on genomic DNA obtained from recipient sow No.0306, donor cells and each of the piglets from recipient sow No.0306. Twelve microsatellite loci (SW353, S0386, S0355, SW72, S0070, SW159, SW2053, SW24, S0107, S0068, SW936, and TNFB) located on different porcine chromosomes were first visualized by 3% agarose gel electrophoresis. Then, microsatellite loci showing different bands were selected and further confirmed by capillary gel electrophoresis with fluorescently labeled amplimers and laser scanning using an ABI 3700 Genetic Analyzer and genemapper 4.0 (Applied Biosystems, Foster City, CA, USA).

To determine the methylation status of the CMV and EF1α promoters, isolated genomic DNA was subjected to bisulfite treatment using CpGenome^™^ Turbo Bisulfite Modification Kit (Millipore, Billerica, MA, USA) according to the kit protocol. After bisulfate treatment, modified DNA was subjected to nested‐PCR (see Table 1 for primer sequences and PCR conditions). The PCR products were purified and cloned into the PGM‐T vector (Tiangen). Finally, at least 10 positive DNA samples were sent to Tiangen Corporation for sequencing.

### Fluorescent protein expression analysis

Isolation of cells from the ear and tail tissues of the piglets was performed as described above. Confluent cultured cells were analyzed under an epifluorescence microscope (Nikon, Tokyo, Japan) equipped with a digital camera. For further confirmation of fluorescent expression, the cells were digested and analyzed using a BD FACSCalibur flow cytometer (Becton Dickinson Immunocytometry Systems) to determine the expression of EGFP and tdTomato fluorescent proteins.

Major tissues were collected from the piglets that died within 3–5 h after death. After embedding in Tissue‐Tek OCT Compound (Sakura Finetek, Tokyo, Japan) at −80 °C overnight, cryosectioning and staining with Hoechst 33342 was performed. Subsequently, the expression of fluorescent proteins was evaluated using a confocal laser‐scanning microscope (Olympus Fv100, Tokyo, Japan).

### Statistical analyses

Data expressed as percentages were compared by chi‐squared test using spss (SPSS Inc., Chicago, IL, USA). A probability of less than 0.05 was considered statistically significant.

## Results

### The proliferation of donor cells

**Figure 1:**
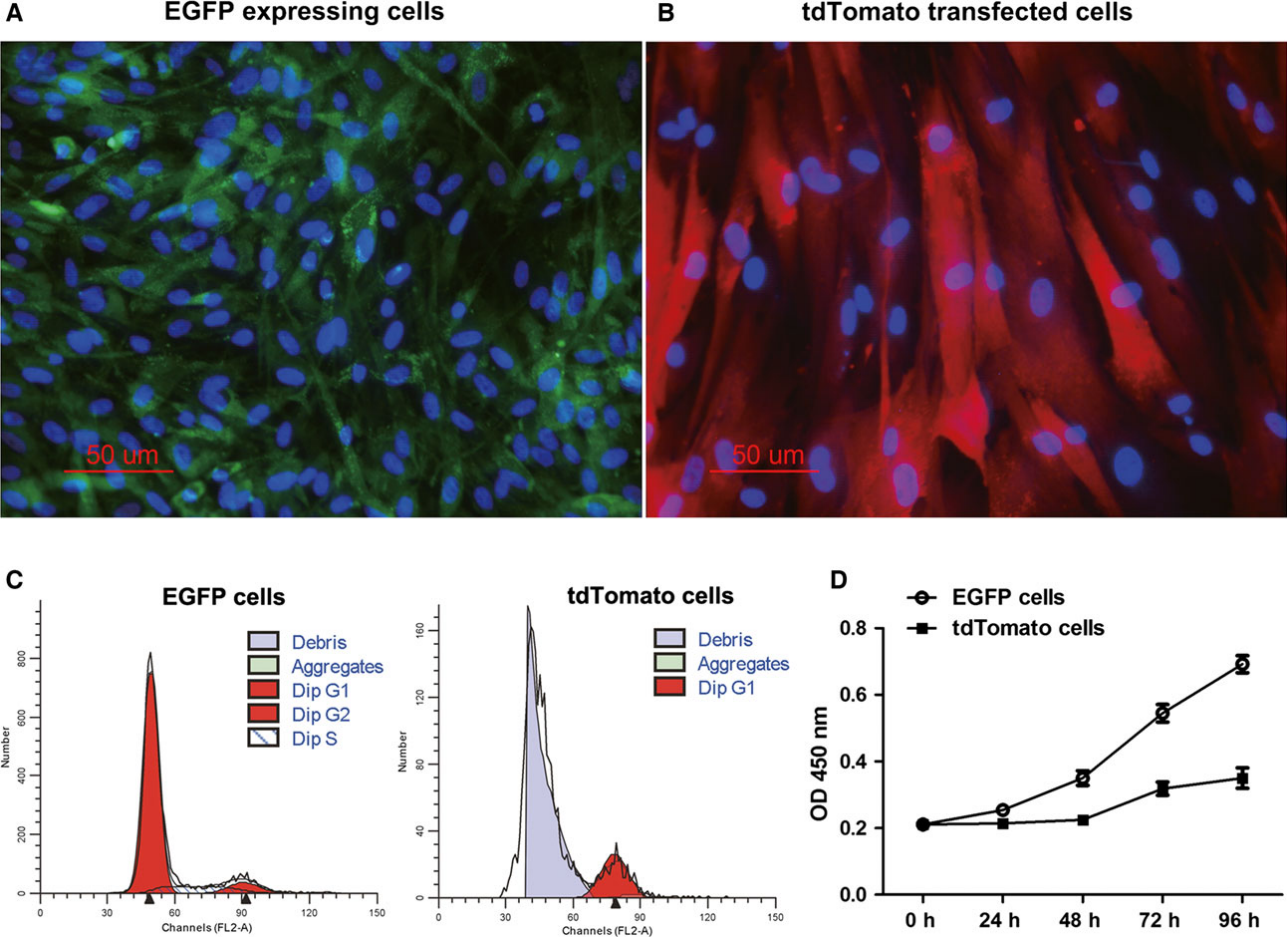
The growth characteristics of cells derived from the same batch of SCNT donor cells for aggregated embryos. (A and B) The morphology of EGFP‐expressing cells and tdTomato‐transfected cells. Cells were stained with Hoechst 33342 (blue). (C) Cell cycle profiles were evaluated by flow cytometry. (D) Results of the cell proliferation assay.

In this study, large white pig fetal fibroblast cells isolated from a male fetus expressing EGFP and Songliao black pig fetal fibroblast cells isolated from a female transfected with tdTomato were used as SCNT donor cells. The EGFP‐expressing cells showed better morphology than the tdTomato‐transfected cells. Most of the tdTomato‐transfected cells had an elongated morphology and large nuclei (Fig. 1A,B). To compare the differences in their growth characteristics, the cell cycle phase and proliferation rates of the EGFP‐ and tdTomato‐expressing cells derived from the same batch of SCNT donor cells were analyzed. Compared with the EGFP‐expressing cells, there was a large amount of cell debris in the tdTomato‐transfected cells, and the nuclei of most of these cells were enlarged (Fig. 1C). The proliferation rate of the tdTomato‐transfected cells was significantly slower than that of the EGFP‐expressing cells (Fig. 1D).

### Birth of piglets with coat color chimerism from aggregated embryos

**Figure 2:**
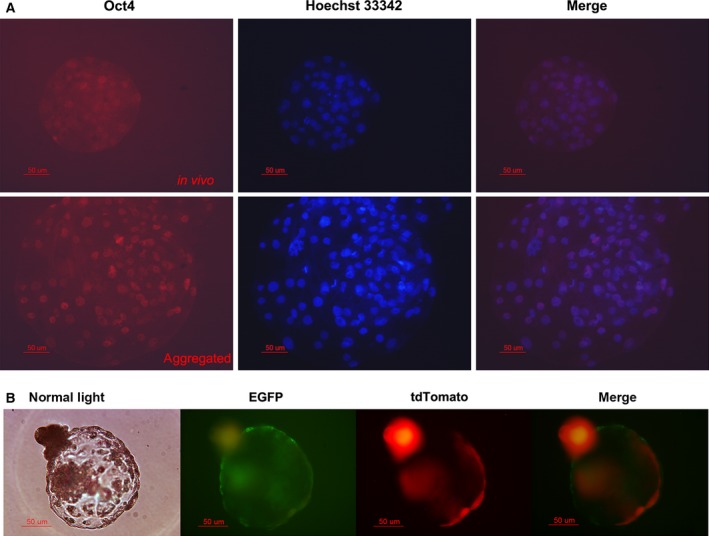
Fluorescence detection in aggregated blastocysts. (A) Oct4 immunostaining between *in vivo* fertilization blastocysts and aggregated blastocysts. (B) The expression of fluorescent proteins in aggregated blastocysts.

To better observe the chimeric ratio, the same number of two kinds of embryos were aggregated. The optimal ratio for aggregating embryos derived from EGFP‐expressing cells and embryos derived from tdTomato‐transfected cells was determined. We observed a higher blastocyst formation rate (72.31%) in the 2 : 2 aggregation groups (Table 2), and this aggregating ratio was used for the following experiment. Fluorescent detection showed that both EGFP and tdTomato fluorescence was detected in the aggregated blastocyst (Fig. 2B). To evaluate the cytological quality of the aggregated blastocyst, immunostaining of Oct4 was compared between *in vivo* fertilization blastocysts and chimeric blastocysts (Fig. 2A). The results revealed that there was no significant difference in Oct4 expression between them.

**Table 2:** Development of aggregated 4‐cell stage embryos

Aggregated ratio	Number of aggregated embryos	Number of aggregated blastocysts (%)
1 : 1	121	39 (32.23)^a^
2 : 2	130	94 (72.31)^b^
3 : 3	108	54 (50.00)^c^

Different superscripts (a, b, c) in the same column denote a significant difference (*P* < 0.05).

**Figure 3:**
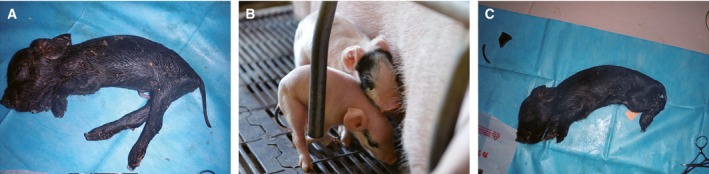
Four piglets delivered by the recipient sow No.0306. (A) 2460, female. (B) Liter (2461), male, black skin was observed in the right eye, on the dorsum and on the tail; Rature (2463), male, black skin was observed on the head. (C) 2462, female.

A total of 260 blastocysts were transferred surgically to the uteri of ten naturally cycling female surrogate sows, with an average number of 26 blastocysts per recipient (Table 3). Four recipients were determined to be pregnant at day 27. Among them, the recipient sow No.0306 delivered two male and two female piglets (Table 4). The two female piglets of No.0306 exhibited entirely black skin color, and both died at birth; the two male piglets (2461 and 2463) showed a chimeric phenotype with black and white skin color (Fig. 3). We renamed 2461 as Liter and 2463 as Rature. Black skin was observed in the right eye, on the dorsum and on the tail of Liter, but only on the head of Rature.

**Table 3:** *In vivo* development of aggregated embryos

Recipient sow No.	No. of embryos transferred	Blastocyst stage (day^a^	Recipient cycle (day)	Day 27 pregnancy detection	Gestation length (day)	Piglets born	Cloning Efficiency^b^
0571	15	Day 6	Day 5	–	–	–	–
0574	17	Day 6, Day 7	Day 4	+	Abortion at Day 29	–	–
0301	26	Day 6	Day 4	+	118	4	15.4%
0302	21	Day 6, Day 7	Day 5	–	–	–	–
0303	21	Day 6, Day 7	Day 6	–	–	–	–
0304	32	Day 6	Day 5	–	–	–	–
0305	37	Day 6, Day 7	Day 4	–	–	–	–
0306	26	Day 6, Day 7		+	115	4	15.4%
0307^a^	35	Day 6		+	Resorption	–	–
0309	30	Day 6, Day 7, Day 8		–	–	–	–
Total	260	–		4	–	8	3.3%

The majority of the transferred embryos were D6 blastocysts.

Cloning efficiency was calculated as follows: No. of piglets/No. of embryos transferred.

a The recipient sow was still pregnant at Day 62.

**Table 4:** Piglets data

Recipient sow number	Piglet number	Body weight at birth	Sex	Skin color
0301	2453	1.40 kg	Male	White
0301	2455	1.36 kg	Male	White
0301	2457	0.68 kg	Male	White
0301	2459	1.56 kg	Male	White
0306	2460	1.30 kg	Female	Black
0306	2461 (Liter)	0.80 kg	Male	Black and white
0306	2462	0.60 kg	Female	Black
0306	2463 (Rature)	1.50 kg	Male	Black and white

### Chimerism in piglets was confirmed by genotypes

**Figure 4:**
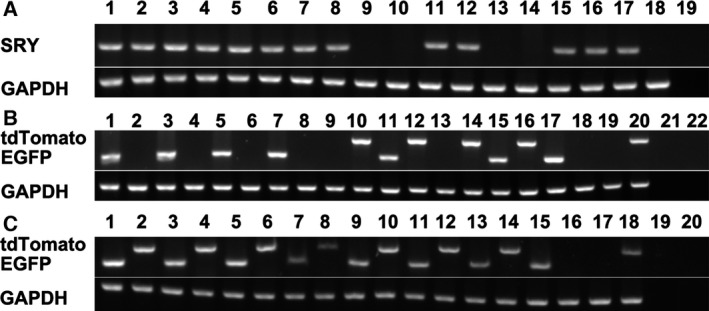
Genotype identification of chimeric piglets by PCR analysis. GAPDH was included as a loading control. (A) SRY gene amplification. Lanes 1, 3, 5, 7, 9, 11, 13, and 15: amplification of genomic DNA from the ears of piglets 2453, 2455, 2457, 2459, 2460, 2461, 2462, and 2463, respectively; lanes 2, 4, 6, 8, 10, 12, 14, and 16: amplification of tail genomic DNA from piglets 2453, 2455, 2457, 2459, 2460, 2461, 2462, and 2463, respectively; lanes 17, 18 and 19: amplification of genomic DNA from EGFP‐expressing cells, tdTomato‐transfected cells and ddH_2_O (negative control), respectively. (B) The EGFP gene and tdTomato gene were amplified from the genomic DNA of newborn piglets. Lanes 1, 3, 5, 7, 9, 11, 13, 15, 17, 19, and 21: the EGFP gene was detected in the ear genomic DNA from piglets 2453, 2455, 2457, 2459, 2460, 2461, 2462, and 2463; EGFP expressing cells; tdTomato‐transfected cells; and ddH_2_O (negative control), respectively; lanes 2, 4, 6, 8, 10, 12, 14, 16, 18, 20, and 22: the tdTomato gene was detected in the ear genomic DNA from piglets 2453, 2455, 2457, 2459, 2460, 2461, 2462, and 2463; EGFP‐expressing cells; tdTomato‐transfected cells; and ddH_2_O (negative control), respectively. (C) The EGFP gene and the tdTomato gene were amplified from genomic DNA from different tissues of Rature. Lanes 1, 3, 5, 7, 9, 11, 13, 15, 17, and 19: the EGFP gene was detected in the genomic DNA from liver, lung, heart, kidney, spleen, skin, testis, EGFP‐expressing cells, tdTomato‐transfected cells and ddH_2_O (negative control), respectively; lanes 2, 4, 6, 8, 10, 12, 14, 16, 18, and 20: the tdTomato gene was detected in the genomic DNA from liver, lung, heart, kidney, spleen, skin, testis, EGFP‐expressing cells, tdTomato‐transfected cells, and ddH_2_O (negative control), respectively.

**Figure 5:**
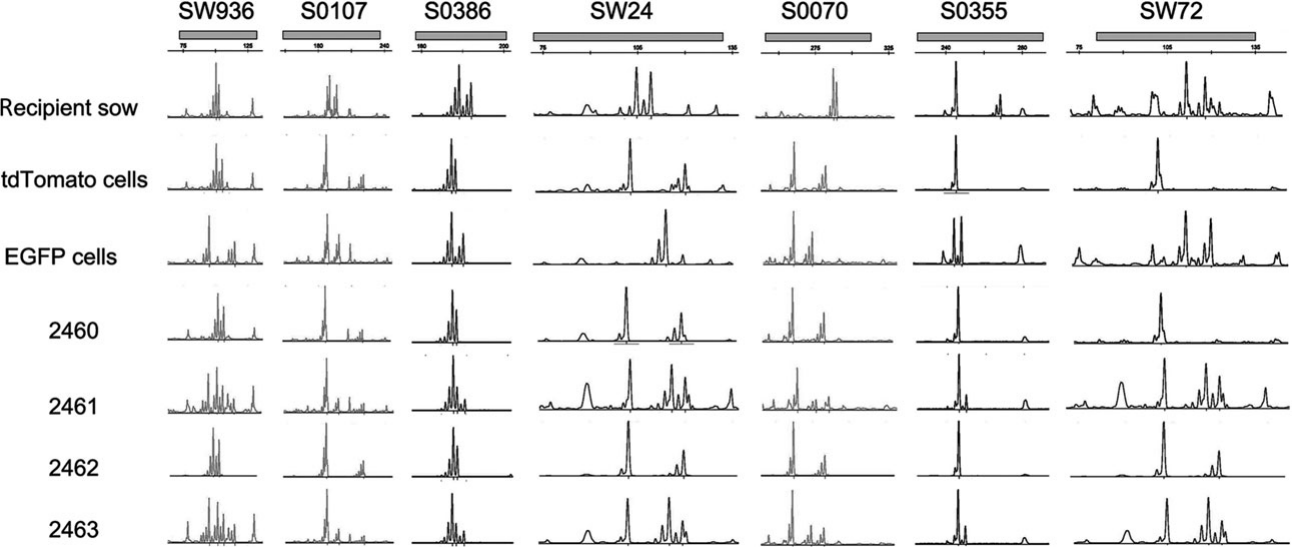
Representative microsatellite loci were analyzed in the recipient sow No.0306, tdTomato‐transfected cells, EGFP expressing cells, 2460, Liter (2461), 2462, and Rature (2463). See also Table 5.

To confirm the chimerism in the genomes of the piglets, their genotypes were determined from extracted genomic DNA. As shown in Fig. 4A, the SRY gene was amplified in the ear and tail genomic DNA from Liter and Rature. Both of the EGFP and tdTomato genes were found in Liter and Rature (Fig. 4B). In addition, the EGFP and tdTomato genes were both dsetected in the genomic DNA collected from all the tested organs from Rature (Fig. 4C). Microsatellite analysis was performed on the genomic DNA from recipient sow No.0306, the donor cells and each of the piglets from recipient sow No.0306. The results confirmed that the genotypes of both Liter and Rature were identical to the composition of the EGFP‐ and tdTomato‐expressing cells but were not identical to the genotype of recipient sow No.0306 (Fig. 5 and Table 5).

**Table 5:** Results of microsatellite examination

Animal	Recipient sow	tdTomato cells	EGFP cells	2460	2461	2462	2463
DNA origin	Ear	Cells	Cells	Ear	Ear	Ear	Ear
Sex	XX	XX	XY	XX	XY	XX	XY
SW936	102/104	102/106	95/115	102/106	95/102/106/115	102/106	95/102/106/115
S0107	188/194	186/220	186/197	186/220	186/197/220	186/220	186/197/220
S0386	179/185	174/175	174/179	173/175	173/175/179	174/175	174/175/179
SW24	104/109	102/119	115	102/119	102/115/119	102/120	102/115/119
S0070	292/294	262/283	262/275	262/283	262/275/283	262/283	262/275/283
S0355	246/269	246	246/250	246	246/250	246	246/250
SW72	112/118	101	111/119	101	101/110/118	102	101/110/118

### Chimerism in EGFP and tdTomato fluorescence expression

**Figure 6:**
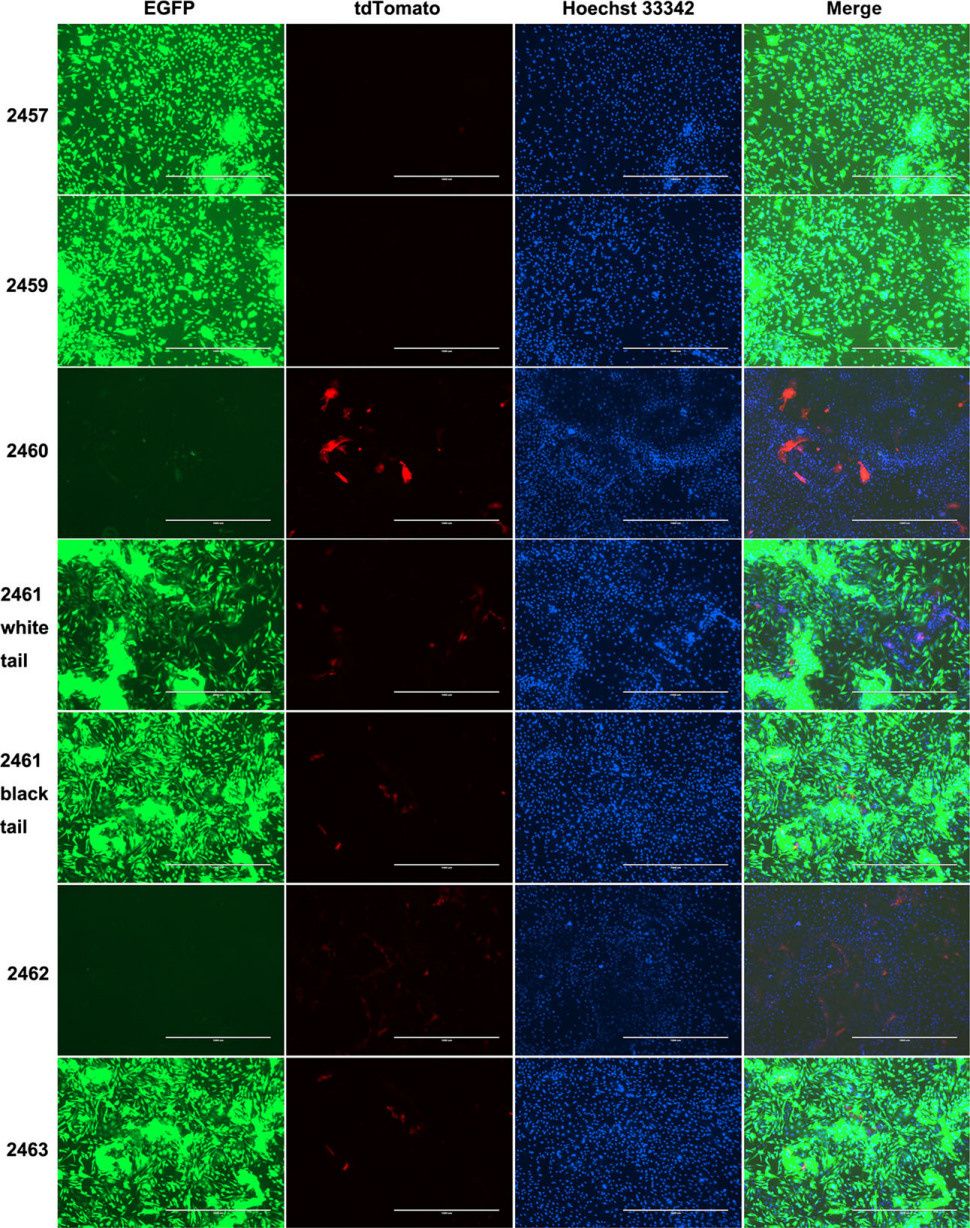
The expression of fluorescent protein in isolated cells. Photographs of Liter (2461) and Rature (2463) were taken by first finding cells with tdTomato fluorescent protein expression. Isolated cells from 2457 and 2459 were used to represent the fluorescent protein expression in piglets from the recipient sow No.0301. The scale bar represents 1000 μm.

**Figure 7:**
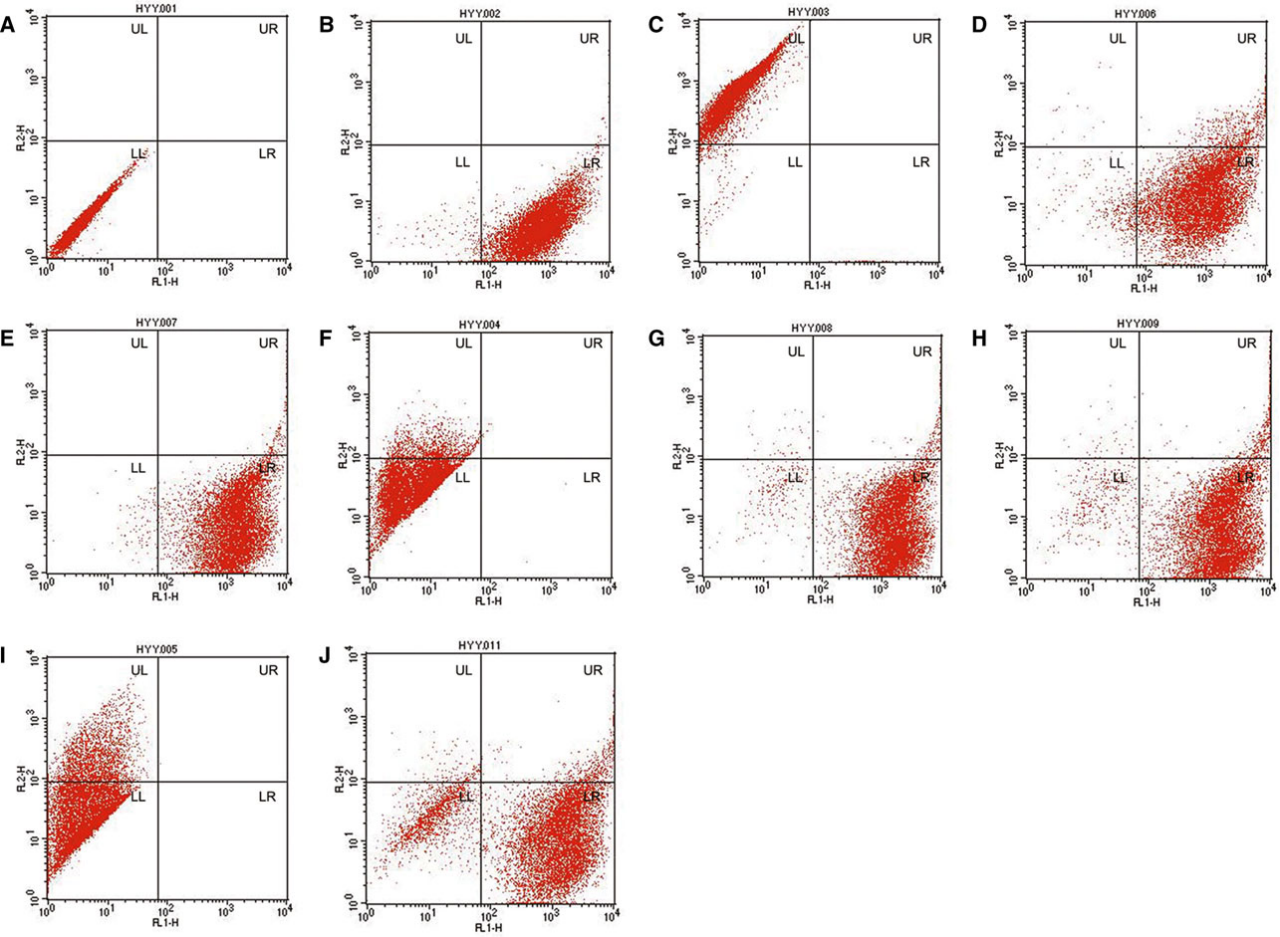
The number of cells with EGFP and/or tdTomato fluorescent protein expression was evaluated by flow cytometry. (A) Control fibroblast cells. (B) EGFP‐expressing cells. (C) tdTomato‐expressing cells. (D) 2457. (E) 2459. (F) 2460. (G) Cells from a part of the tail of piglet 2461(Liter) with white skin color. (H) Cells from a part of the tail of piglet 2461 with black skin color. (I) 2462. (J) 2463 (Rature). (D–J) Cells were all isolated from the tail tissues of the newborn piglets. See also Table 6.

**Figure 8:**
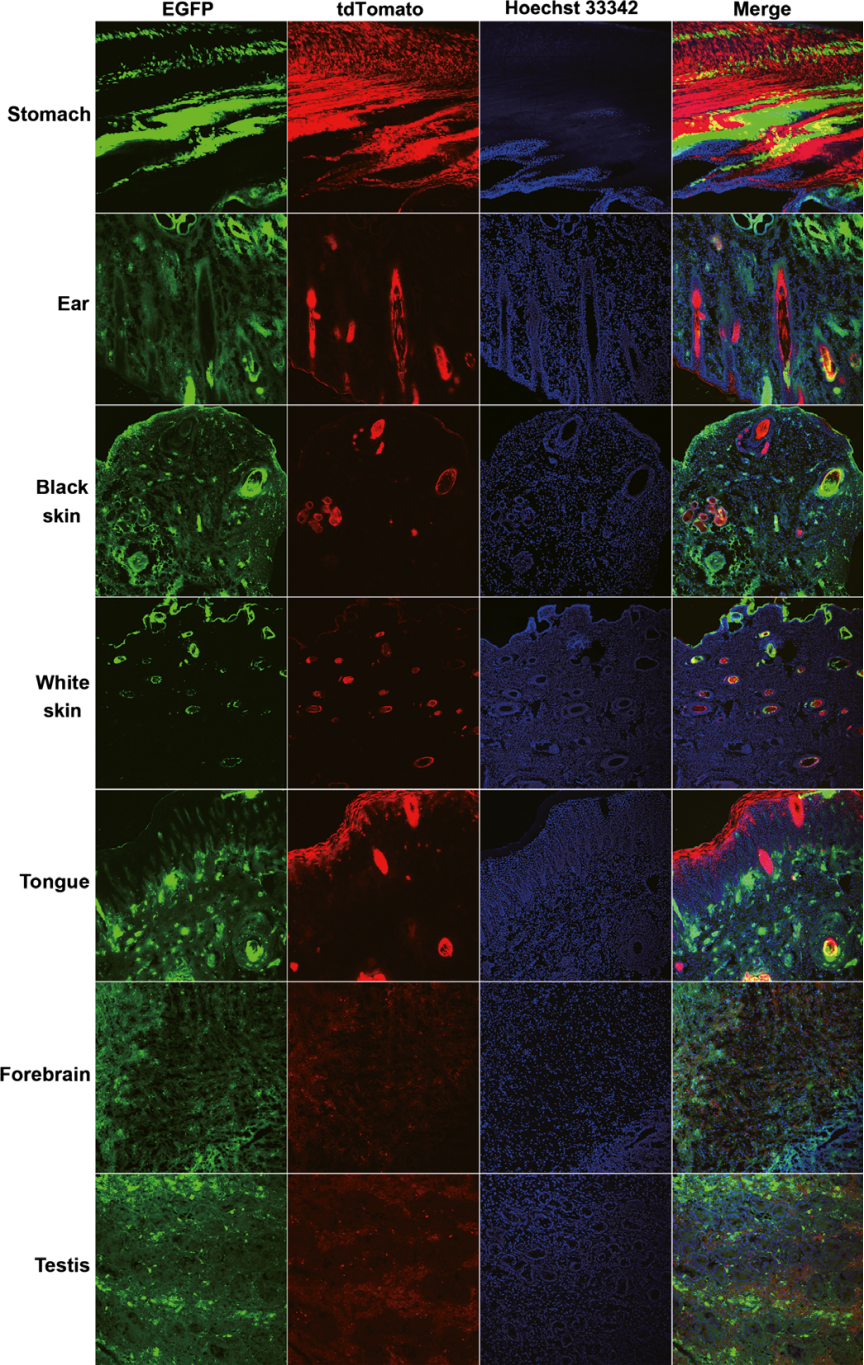
Tissues from Rature with obvious co‐expression of EGFP and tdTomato fluorescent protein. Photographs were taken by finding cells with tdTomato fluorescent protein expression.

**Figure 9:**
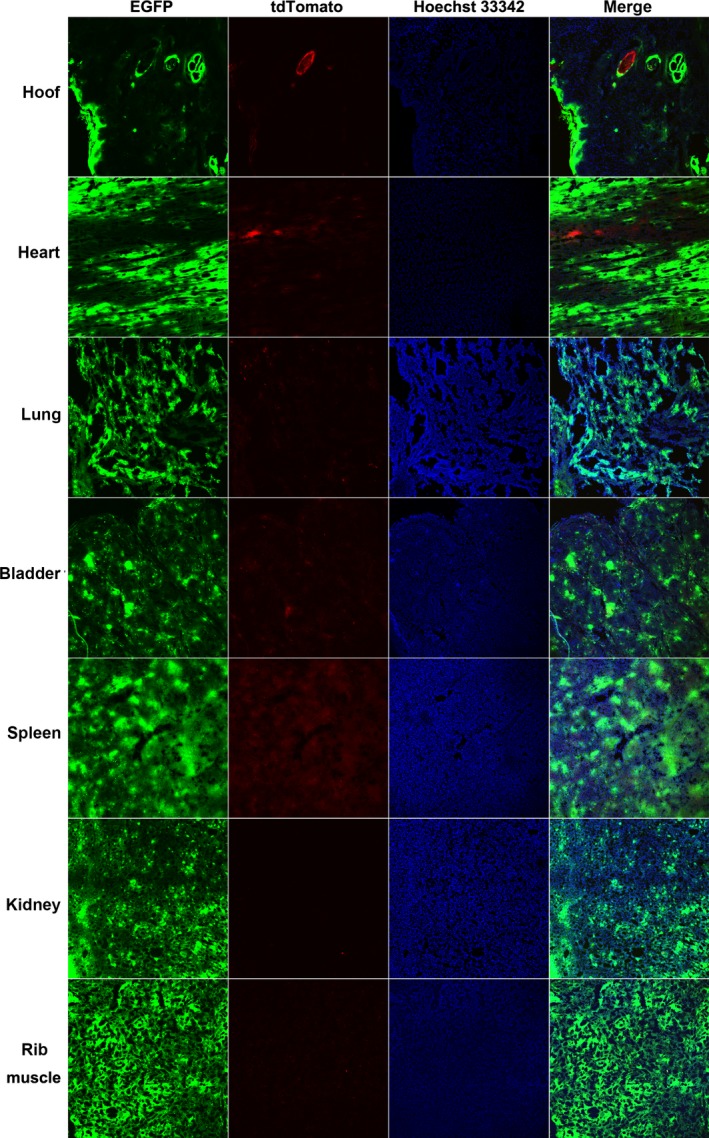
Tissues from Rature with low tdTomato fluorescent protein expression. Photographs were taken by first finding cells with tdTomato fluorescent protein expression.

**Figure 10:**
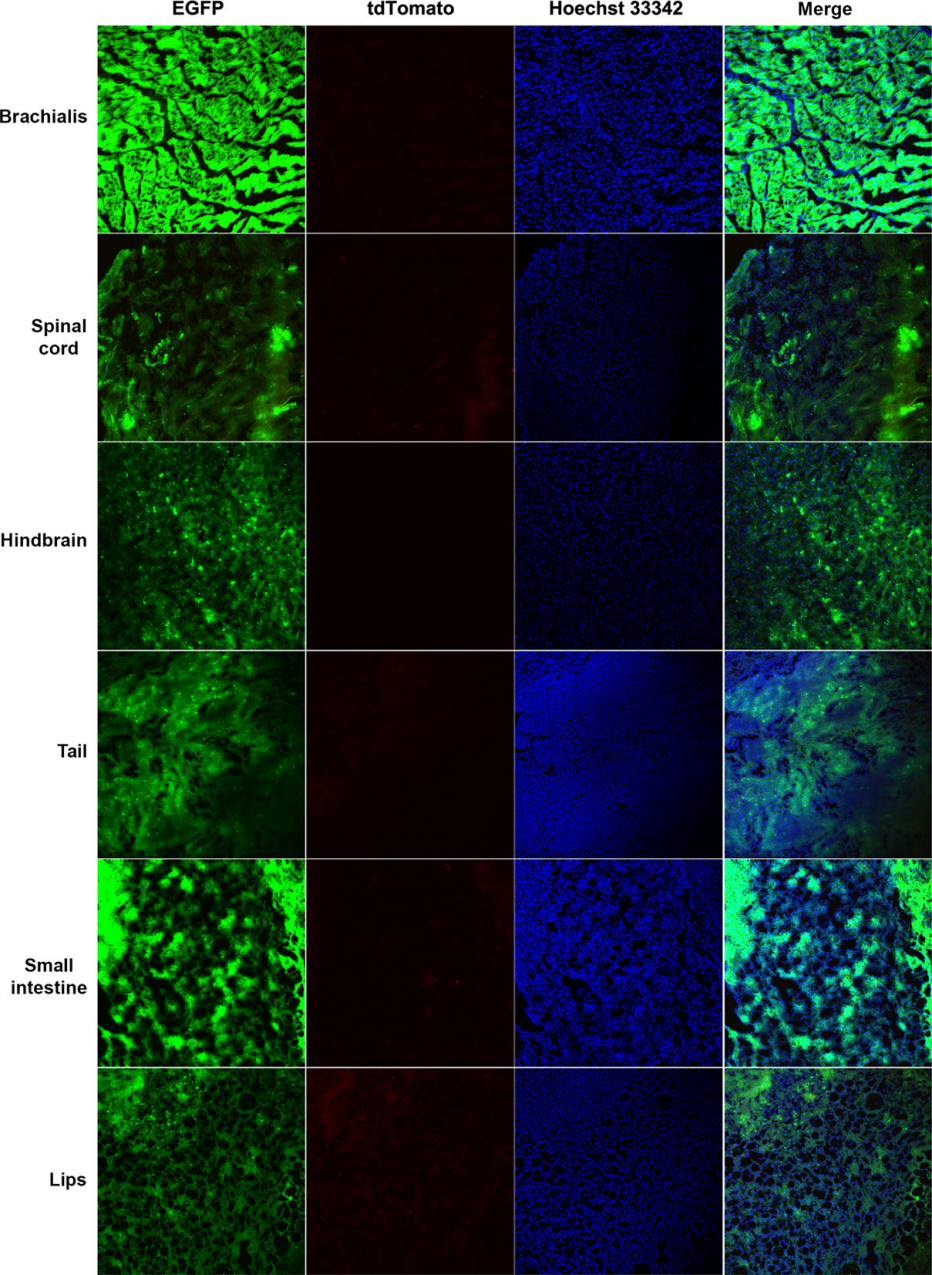
Tissues from Rature with very low tdTomato fluorescent protein expression. Photographs were taken by first examining cells with tdTomato fluorescent protein expression.

Cells isolated from the tail tissues of the newborn piglets were observed under an epifluorescence microscope. The EGFP fluorescent protein and the tdTomato fluorescent protein were both observed in the isolated cells of Liter and Rature; however, the tdTomato fluorescent protein was observed in only a small number of the isolated cells (Fig. 6). The results of flow cytometry analysis confirmed that the tdTomato fluorescent proteins only existed in a small portion of the cells isolated from Liter and Rature (Fig. 7). As the tail of Liter can be divided into two parts according to skin color, we isolated cells from these two parts individually. The isolated cells were classified as black tail‐derived cells or white tail‐derived cells according to the skin color. Expectedly, the number of cells expressing tdTomato fluorescent protein was low but did not vary largely between the black tail‐ and white tail‐derived cells (Figs 6 and 7). The fluorescent proteins of major tissues of Rature were evaluated by confocal microscopy. Overall, EGFP‐positive cells dominated in most tissues, and tdTomato‐positive cells were clearly observed in some tissues (Figs 8, 9 and 10). As the skin had both white and black colored sections, we classified the isolated skin tissues as either black skin or white skin according to their skin color. Similar to the above results, there were low amounts of tdTomato‐positive cells among them without considering the different skin colors (Fig. 8). In addition, the chimerism could also be detected in the forebrain and testis (Fig. 8).

### Methylation status of the CMV and EF1α promoters in chimeric pigs

**Figure 11:**
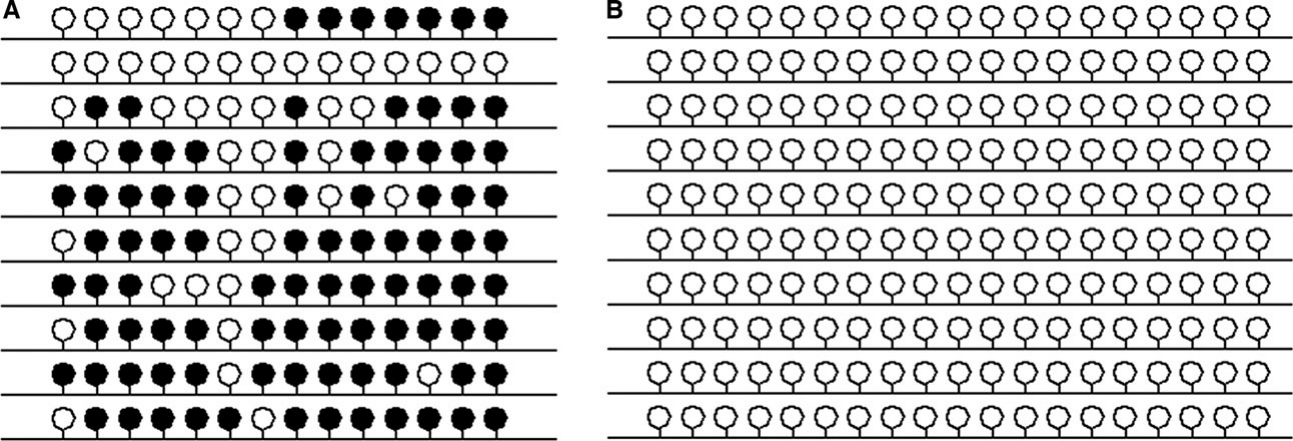
Results of bisulfite sequencing. (A) Methylation status of the CMV promoter. (B) Methylation status of the EF1α promoter. A filled black circle represents a methylated CpG dinucleotide, and an open circle represents an unmethylated CpG dinucleotide.

The above results indicated that there were low amounts of tdTomato‐positive cells, and some cells derived from the cloned piglets had neither EGFP nor tdTomato fluorescent protein expression. It is well known that the CMV promoter is one of the most commonly used promoters for forcing the expression of transgenes in mammalian cells. However, many publications have documented that the CMV promoter would be gradually silenced over a long period of culturing. It was hypothesized that the CMV promoter might be methylated during development. Therefore, we examined the methylation status of the EGFP gene EF1α promoter and the tdTomato gene CMV promoter in the genomic DNA collected from Rature's ear (Fig. 11). The results revealed that the CMV promoter was highly methylated (92/140, 65.7%). However, there was no methylation of the EF1α promoter. These results may explain the silencing of fluorescent protein expression in some cells.

**Table 6:** Results of two‐color flow cytometry assay

Cell type	UL (% total)	UR (% total)	LL (% total)	LR (% total)
Control	0	0	100	0
EGFP‐expressing cells	96.88	0.01	2.5	0.61
tdTomato‐transfected cells	0	0.18	1.49	98.33
2457 tail	0.16	5.94	3.59	90.31
2459 tail	0	1.3	0.93	97.77
2460 tail	10.91	0.1	88.97	0.02
2461 white tail	0.35	1.92	2.42	95.31
2461 black tail	0.36	3.51	3.77	92.36
2462 tail	24.04	0.01	75.95	0
2463 tail	1.28	4.17	15.9	78.65

### Influence of fluorescent proteins on the proliferation of bladder cancer cells

**Figure 12:**
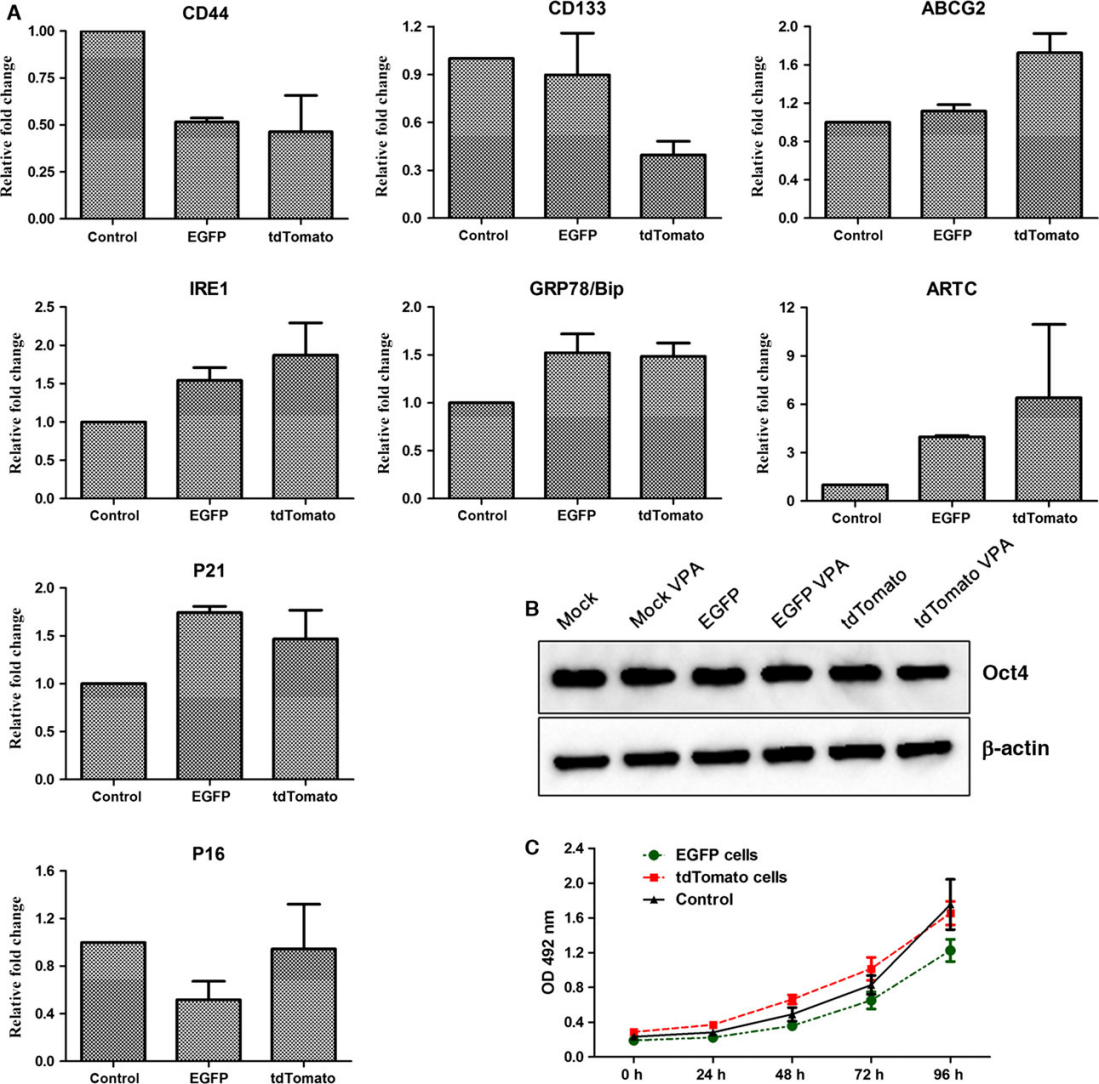
(A) Gene expression levels in EGFP expressing and tdTomato‐transfected UC5 cells were determined by quantitative PCR. (B) Expression of the Oct4 protein in EGFP‐ expressing and tdTomato‐transfected UC5 cells treated with or with VPA. β‐actin was used as a loading control. (C) Cell proliferation of EGFP‐expressing and tdTomato‐transfected UC5 cells was determined by MTT assay.

**Figure 13:**
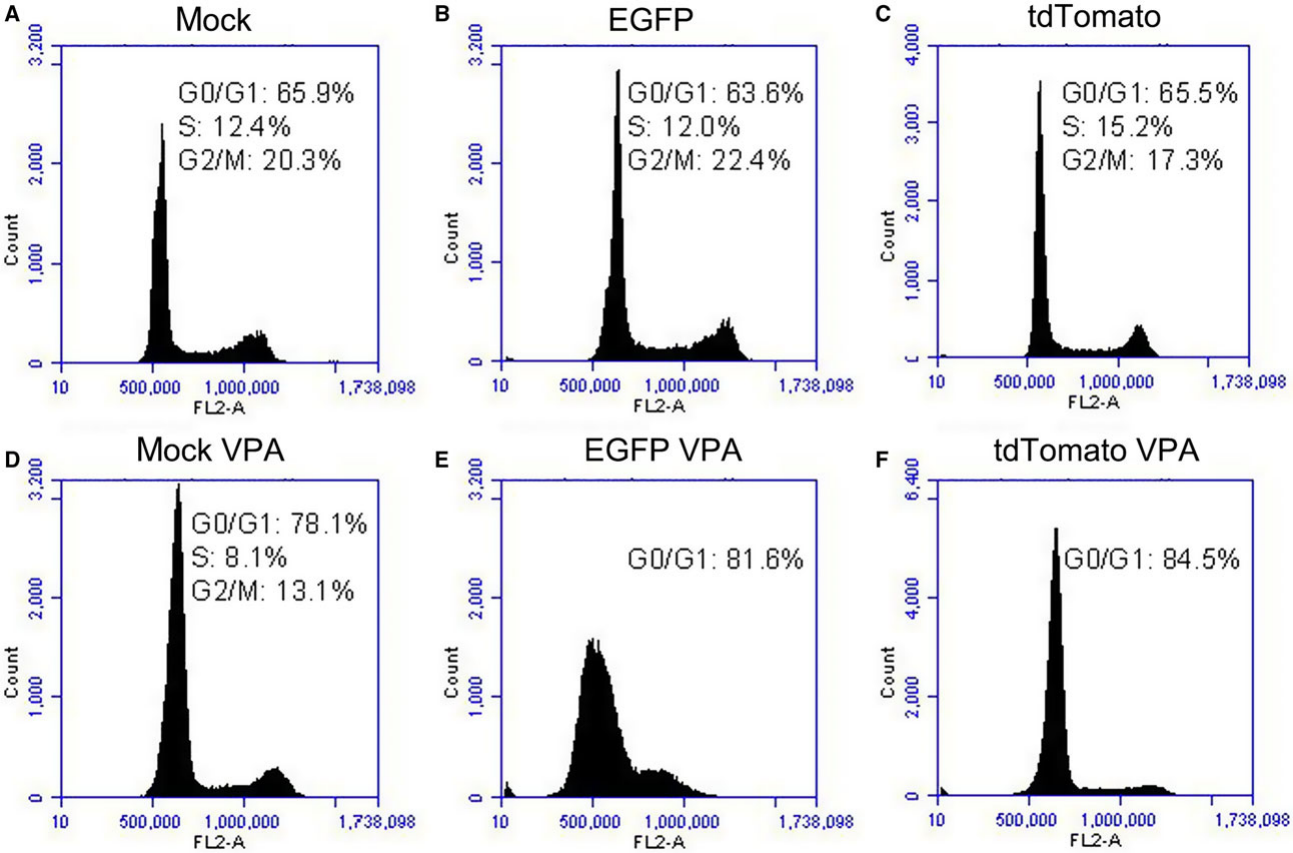
Cell cycle distribution of EGFP‐expressing and tdTomato‐transfected UC5 cells revealed by flow cytometry.

Alternatively, the inconsistent distribution ratio of tdTomato fluorescent protein‐expressing cells and EGFP fluorescent protein‐expressing cells in chimeric pigs may be due to the different impacts of these proteins on cell growth. To determine the impacts of fluorescent proteins on cell growth, the blank vector, the pCMV‐EGFP vector and the pCMV‐tdTomato vector were transfected into the bladder cancer cell line UC5 and selected with 700 μg·mL^−1^ of G418. The MTT assay results indicated that there were similar growth curves among the control‐, EGFP‐, and tdTomato‐transfected UC5 cells (Fig. 12C). The cell cycle tests also showed similar cell cycle distributions among these three cell lines (Fig. 13). However, the colony‐formation rate of the tdTomato‐transfected UC5 cells was lower than that of the EGFP cells in the soft agar colony formation assay (6.83 ± 0.32% vs. 8.83 ± 0.32%, respectively, *P* = 0.018). Therefore, we attempted to exploit the underlying mechanism of this phenomenon.

The expression levels of the cancer stem cell‐specific markers CD44, CD133, and ABCG2, the tumor suppressor genes p21 and p16, and the endoplasmic reticulum (ER) stress‐related genes IRE1, GRP78/BIP, and ARTC were determined by quantitative PCR (Fig. 12A). As shown in Fig. 12, CD44 and CD133 were expressed at low levels in tdTomato‐transfected UC5 cells. The expression of P21 was up‐regulated in both EGFP‐ and tdTomato‐transfected UC5 cells, while the expression of P16 was down‐regulated in EGFP‐transfected UC5 cells. The expression levels of the IRE1, GRP78/BIP, and ARTC genes were up‐regulated in both fluorescent protein‐expressing cells, particularly in tdTomato‐transfected UC5 cells.

The effects of the fluorescent proteins on resistance to anticancer agents were also examined, and valproic acid (VPA) was applied in this study. First, the survival rates of fluorescent protein‐expressing cells with VPA treatment were determined by flow cytometry. The results showed that 44.95 ± 2.76% of the tdTomato‐transfected UC5 cells survived after 5 mm VPA treatment for 48 h, while the survival rate of EGFP‐transfected UC5 cells was 50.45 ± 0.92% (*P* = 0.000). Furthermore, the cell cycle tests also showed that a great mass of cells was blocked at the G0/G1 phase in response to VPA treatment, especially in the tdTomato‐expressing cells (Fig. 13). These results indicated that tdTomato‐expressing cells may be more sensitive to environmental stress. However, the expression of the Oct4 protein was only slightly down‐regulated in the tdTomato cells treated with 5 mm VPA for 48 h (Fig. 12B).

## Discussion

In this study, porcine chimeras were produced by aggregating SCNT embryos at the 4‐cell stage. The composition rates between the EGFP‐ and tdTomato‐expressing cells varied greatly, and several factors could contribute to this outcome.

Many studies have revealed that the development of somatic cell nuclear transfer (SCNT) embryos can be affected by some characteristics of their donor cells 22, 23, 24, 25. In this study, the EGFP‐expressing cells were freshly isolated fetal fibroblast cells, and the tdTomato‐expressing cells were transfected cells. It is possible that the high passage number of the transfected cells could cause the health of the cells to be worse than that of the freshly isolated fetal fibroblast cells. In fact, most of the tdTomato‐transfected cells had a more elongated morphology and larger nuclei but lower proliferation rates than the EGFP‐expressing cells. However, there was no significant difference in the expression of Oct4 between chimeric and *in vivo* fertilization blastocysts, suggesting that the cytological quality of the embryos did not vary greatly after aggregation. Oct4 is critical for the development of blastocysts, and normal blastocysts cannot form in the absence of Oct4 26.

Furthermore, DNA methylation of the CMV promoter is likely responsible for the low expression of the tdTomato fluorescent protein. It has been suggested that transgene copy number, DNA methylation and some other factors may affect transgene silencing in mice 27, 28. In this study, the results of bisulfate sequencing were consistent with a previous report that the CMV promoter was marked by DNA methylation 29. However, it should be noted that, even if all of the cells without fluorescent protein expression were considered to be tdTomato cells, tissues were primarily composed of EGFP‐expressing cells. These results indicate that the methylation of the CMV promoter only partially explains the low composition of tdTomato protein‐expressing cells.

The expression of fluorescent proteins would alter the expression of some other genes, such as ER stress‐related genes. ER stress is important for the regulation of apoptosis 30. Negative effects of ER stress have been documented on the early development of bovine SCNT embryos 31. Such alternations in gene expression would lead to the tdTomato‐expressing cells dominating a disadvantageous position in chimeric development. In addition, there may be some toxicity associated with the red fluorescent protein 32. However, considering that two piglets derived from tdTomato‐transfected cells reached full‐term development, the subtle toxicity may not terminate development but may be responsible for the observation that these cells were at a disadvantageous position during chimeric development. As shown in bladder cancer cells, tdTomato protein expression led to these cells being inclined to undergo apoptosis with a lower clone formation rate.

The two chimeric pigs were both male, but there were both EGFP‐ and tdTomato‐expressing cells in the testis. This is consistent with the previous report that the chimera would develop as a male when male and female embryos were combined 11, indicating that the chimera could possibly be an excellent model to study spermatogenesis and infertility. In the future, it would also be convenient to produce xenogeneic organs through the aggregation of embryos with feasible and acceptable modifications. In fact, recently, the apancreatic phenotype was rescued by blastocyst complementation through the injection of blastomeres of normal morula‐stage donor embryos into pancreatogenesis‐disabled embryos in pigs 12. The generation of one “giant” embryo by aggregation of four different embryos can lead to disturbances in the processes of polarization of blastomeres. This can result in impaired processes of morula compaction as well as cavitation during blastocyst formation and resultant embryo morphogenesis. Therefore, investigations of the disturbances in the process of polarization of blastomeres with different transgene modifications would be interesting. In conclusion, aggregating embryos with different transgenetically modified blastomeres could play an important role in future research.

## Author contributions

B.W., L.L., H.O., and D.P developed the concept and designed the experiments. Y.H., Z.L., A.W., X.H., Y.S., L.Y., and T.L. performed the experiments. Y.H. wrote the paper.
